# Metastatic renal cell carcinoma to the oral cavity as first sign of disease: A case report

**DOI:** 10.1002/ccr3.2923

**Published:** 2020-07-14

**Authors:** Shalizeh Patel, Juliana Barros, Ngozi N. Nwizu, Kalu U. E. Ogbureke

**Affiliations:** ^1^ Department of Restorative Dentistry & Prosthodontics School of Dentistry at Houston University of Texas Health Science Center at Houston Houston TX USA; ^2^ Department of Diagnostic & Biomedical Sciences School of Dentistry at Houston University of Texas Health Science Center at Houston Houston TX USA

**Keywords:** Guillain‐Barre syndrome, metastasis, oral cavity, renal cell carcinoma

## Abstract

Renal cell carcinoma metastasis to the oral cavity is rare. Significantly, the oral lesion in this case was the first indication of a malignant disease in the patient. This case underscores the importance of detailed history taking, interpretation of clinical finding, and high index of suspicion for metastatic disease to the oral cavity.

## Graphical abstract

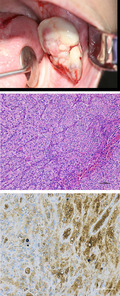

## 1 INTRODUCTION

Metastasis from distant organs, outside the head and neck region, accounts for about 1% of all malignancies of the oral cavity.^1^, ^2^, ^3^, ^4^ In order of frequency, metastatic breast carcinomas to the oral cavity rank highest, followed by lung and kidney cancers.^5^, ^6^ Metastasis develops in approximately one‐third of renal cell carcinomas (RCC), and approximately one‐half of these are distant metastases following an initial primary site diagnosis.^6^ Reports of metastatic renal cell carcinoma to the oral cavity presenting as first sign of disease are rare and indicate very poor prognosis.^7^ Here, we present a case of metastatic RCC to the left buccal mucosa of a 59‐year‐old woman, which represented the first sign of the disease.

## 2 CASE HISTORY

**Figure 1:**
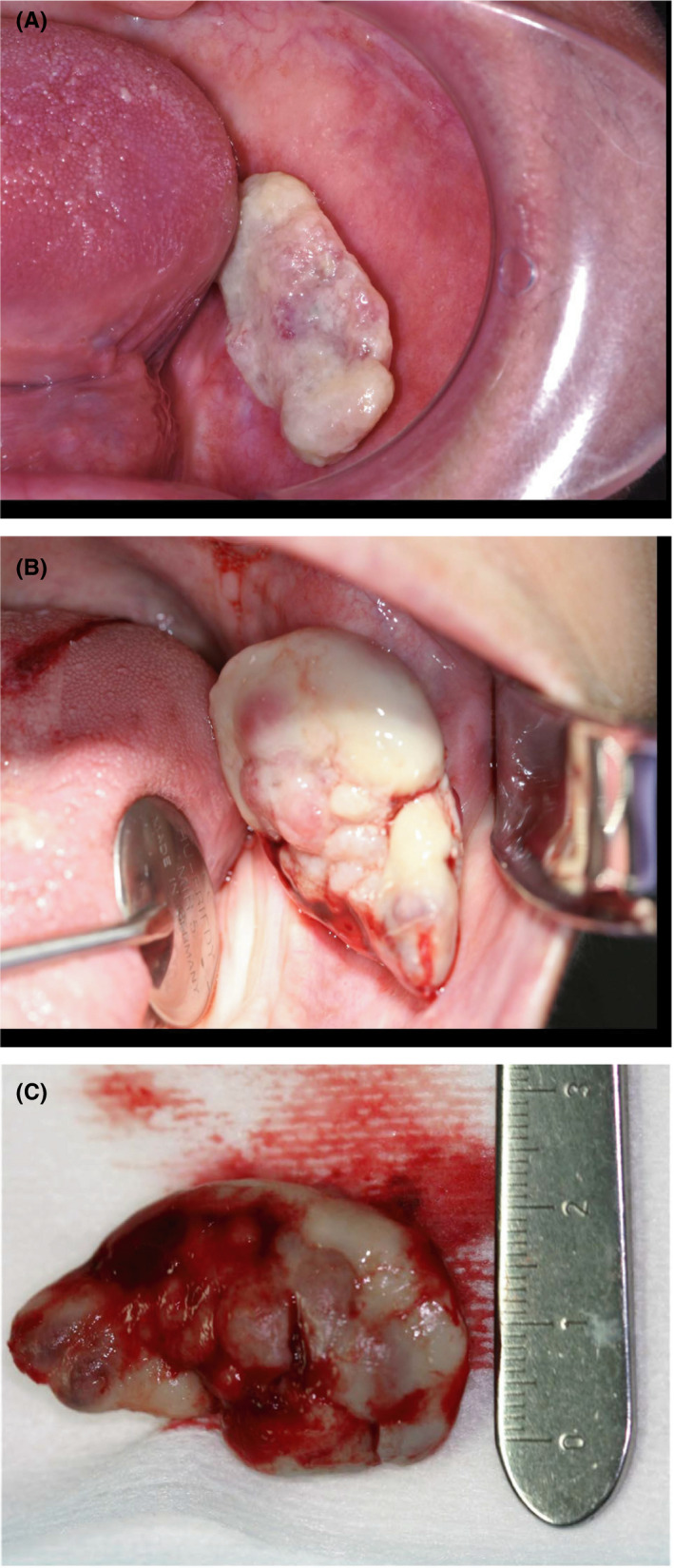
Clinical presentation of intraoral lesion. A, Initial presentation left buccal mucosal lesion with ulcerated and pseudomembranous surface attached to the buccal mucosa by pedunculated stalk. B, Presentation at time of surgery 4 wk later. C, Gross specimen following surgical removal

A 59‐year‐old woman presented at the University of Texas Health Science Center at Houston School of Dentistry clinic with the complaint of a swelling of the left buccal mucosa that was interfering with the fit of her dentures. The patient indicated that she noticed the lesion about three weeks prior to presentation. Past medical history was significant for hip and back pain, and a diagnosis of Guillain‐Barre syndrome as a child. Furthermore, the patient's right kidney was removed along with a “benign” kidney mass in 2014. Intraoral examination revealed an edentulous patient with a pink‐red, oval, ulcerated lesion with a white pseudomembranous surface measuring approximately 38 mm × 25 mm × 17 mm attached to the left buccal mucosa via a pedunculated stalk (Figure 1A). No regional lymphadenopathy was apparent, and a complete head and neck examination was otherwise unremarkable. Other remarkable findings included a significantly elevated blood pressure of 180/100 and a pulse rate of 90. At the time of presentation, the patient was not under the care of a physician.

## 3 DIFFERENTIAL DIAGNOSIS

Based on the clinical features of the lesion, the following differential diagnoses were generated: pyogenic granuloma, buccal fat pad herniation, traumatic ulcerative granuloma, squamous cell carcinoma, and metastatic disease. Subsequently, the patient was scheduled for surgical excision of the lesion, subject to adequate control of her blood pressure.

## 4 OUTCOME

**Figure 2:**
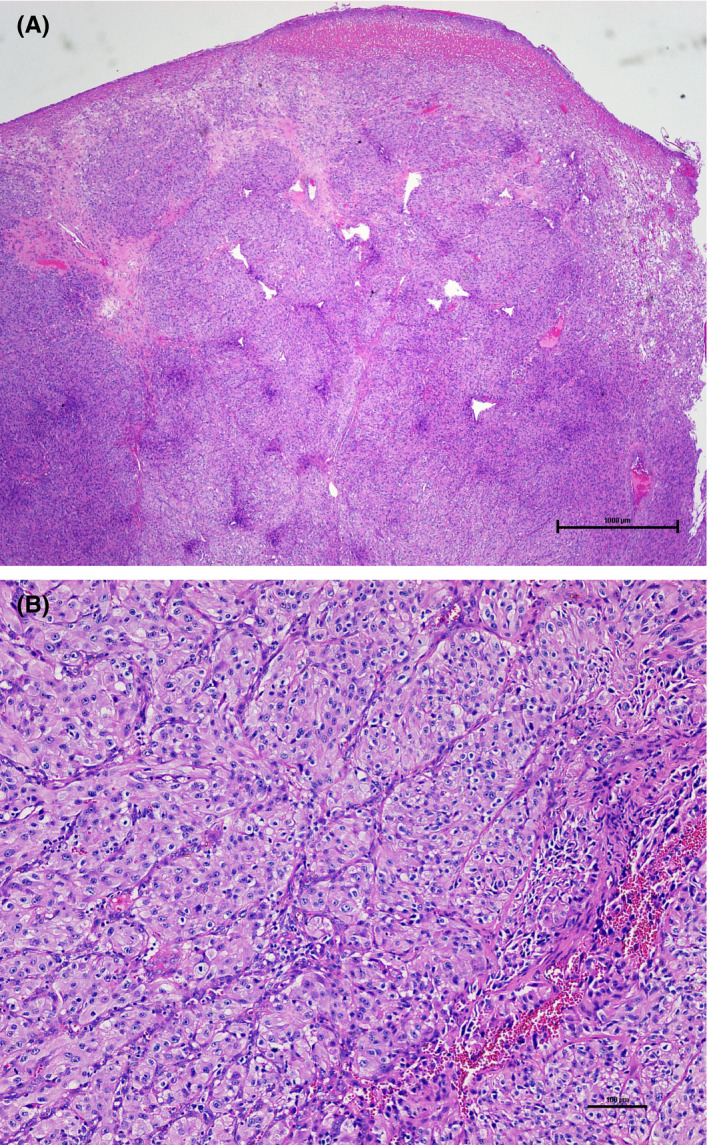
Hematoxylin‐and‐eosin–stained histologic sections of excised specimen. A, Low power (4X) shows sheets of clear cells demarcated by thin fibrous septa and ulcerated surface. B, High power (40X) shows clear cells with pleomorphic renal cell carcinoma cells

**Figure 3:**
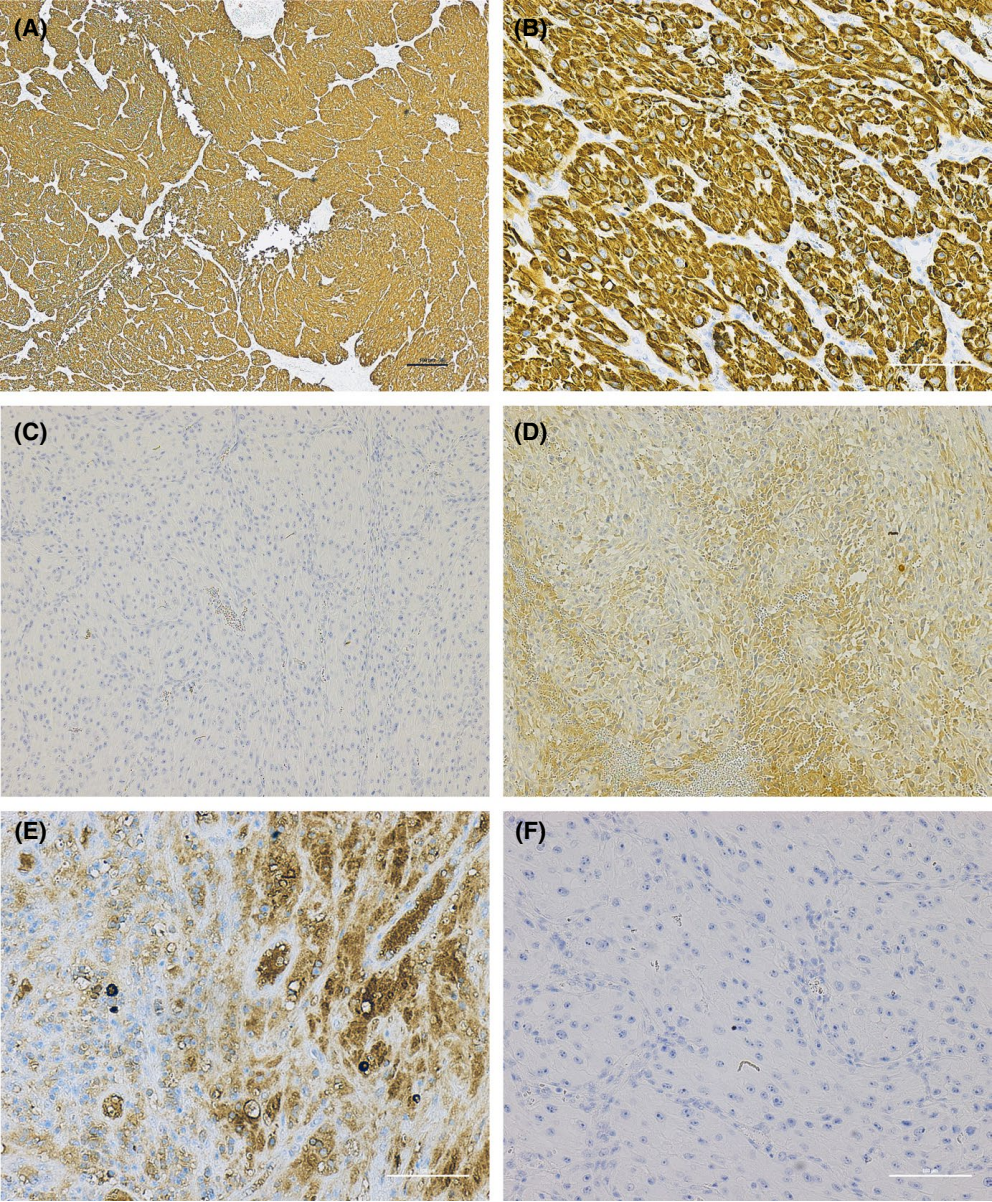
Immunohistochemistry exhibiting diffuse positivity for pan cytokeratin AE1/AE3 (A = 4X; B = 20X); RCC antigen (D = 4X; E = 20X); and (C = 10X) and (F = 20X) representative IgG‐negative controls

At the time of surgery, 4 weeks after the initial presentation, the lesion had increased in size to approximately 50 mm × 30 mm× 25 mm (Figure 1B). A complete excision of the buccal mucosa lesion was achieved, following local anesthetic infiltration, and the specimen (Figure 1C) submitted for histopathologic examination. Histologic examination of the specimen revealed an ulcerated surface mucosa, showing organoid nests of polygonal cells partitioned by fibrovascular septa and delicate capillary‐sized vascular channels, imparting an alveolus‐like formation in areas (Figure 2). Tumor islands were infiltrative with prominent areas of central necrosis and hemorrhage. The malignant cells exhibited features of pleomorphism, hyperchromatism, prominent nucleoli, and increased and abnormal mitoses, with notable cytoplasmic clearing (clear cell morphology). Immunohistochemistry, with appropriate positive and negative controls, was positive for AE1/AE3 and renal cell carcinoma (RCC) antigen (Figure 3). However, S‐100 (not shown) showed punctate positivity, while Melan‐A, myogenin, HMB‐45, chromogranin were negative (not shown). The characteristic histopathologic features in combination with the immunohistochemistry findings confirmed a definitive diagnosis of metastatic RCC. The Table 1 summarizes the results of the immunohistochemistry panels investigated.

**Table 1:** Immunostains used to arrive at a definitive diagnosis

Tumor markers	Immunohistochemistry result
AE1/AE3	+
S‐100	(+)
Melan‐A	−
HMB‐45	−
Myogenin	−
Renal cell carcinomas	+
Chromogranin	−
Synaptophysin	−

Note

Key: + = positive; (+) = punctate positive; and − = negative.

Following a histologic diagnosis, the patient was referred to a specialist hospital for further consultations and management. It was determined that the patient already had disseminated disease to distant organs, including metastasis to the brain. Unfortunately, the patient died from widely disseminated disease few months later.

## 5 DISCUSSION

A salient histopathologic feature of the present case is a distinct and dominant clear cell feature warranting consideration of a constellation of differential diagnosis of clear cell neoplasms of the oral cavity. These include primary and malignant neoplasms of the salivary glands such as mucoepidermoid carcinoma (MEC), clear cell oncocytoma, acinic cell carcinoma (ACC), epithelial myoepithelial carcinoma (EMC), clear cell carcinoma not otherwise specified (CCC‐NOS), and hyalinizing clear cell carcinoma (HCCC). Other differential diagnosis considerations are alveolar soft part sarcoma (ASPS), paragangliomas, and metastatic renal cell carcinoma.

Clear cell differentiation is a common feature of MECs of the salivary gland and occasionally a dominant feature.^8^ Usually, positivity of tumor cells of MECs to MUC5AC immunostain serves to distinguish it from other clear cell tumors.^9^ In clear cell variants of ACCs, tumor cells are positive for diastase‐treated periodic acid‐Schiff (PAS) stain..^10^ Clear cell variants of oncocytoma consists of sheets of oncocytic cells, often with focal areas of typical oncocytic cells that provide requisite clue to their diagnosis.^11^

Although most EMCs are comprised mostly of clear cells, its biphasic morphology comprising intercalated duct‐like structures surrounded by clear myoepithelial cells serves to distinguish it from other clear cell neoplasms of salivary glands.^12^ On the other hand, while HCCC and CCC‐NOS, like EMC, may present with dominant clear cells, HCCC and CCC‐NOS lack the biphasic characteristics of EMC and, instead, show infiltrating thin cords of clear tumor cells. In addition, HCCC shows a densely hyalinizing stroma.^13^

ASPSs are extremely rare in the oral cavity, usually involving the tongue. The histopathologic features of ASPS show uniform, organoid arrangement of cell nests demarcated by fibrovascular septa.^14^ Markedly, ASPS shows typical dishesiveness of the tumor cells and is negative for cytokeratin immunohistochemistry.^14^ Although clear cell changes are not dominant histologic features of paraganliomas, they occasionally are notable. By virtue of their content of “chief cells,” paragangliomas are distinguishable from other clear cell neoplasm by their immunohistochemical reactivity to synaptophysin and chromogranin.^15^

The histopathologic features of RCC are often characteristic and highly suggestive. Furthermore, features of secondary tumors closely mirror those of their primary counterpart. As described above, salient histopathologic features of the present case include a distinctly vascular lesion with characteristic organoid and clear cell morphology, and a pink to clear cytoplasm (Figure 2). A diagnosis of metastatic RCC was confirmed by positive immunohistochemistry for pan keratin AE1/AE3 and renal cell carcinoma (RCC) antigens (Figure 3).

Metastasis of malignant tumors to the oral cavity represent only about 1% of oral malignancies, and in 23% of these cases, the metastatic diseases are the first indication of an unknown primary.^7^, ^16^, ^17^, ^18^, ^19^ Renal cell carcinoma is the second most common primary source of metastatic soft tissue malignancies in the oral cavity in men.^7^, ^20^ The prognosis of metastatic renal cell carcinoma is generally poor, and metastasis to the oral cavity often signals a concurrent widespread disease to other distant organs.^7^ Within the oral cavity, metastasis to the tongue and mandible is much more common compared with other intraoral sites, including the buccal mucosa.^7^, ^17^, ^18^, ^19^, ^20^ As with other metastatic soft tissue malignancies to the oral cavity, metastatic RCC to the oral cavity presents a twofold challenge: recognizing the lesion as a metastasis and determining the primary site. For this reason, metastatic RCC to the oral cavity, often presenting as an exophytic mass, with or without symptoms, leads to a constellation of the differential diagnoses listed above. A biopsy (incisional or excisional) is therefore necessary to establish a diagnosis.

The mechanism of RCC metastasis to the oral cavity is postulated to occur via arterial and paravertebral venous routes (Batson's plexus) rather than the lymphatic system, bypassing the filtration system and enabling the dissemination of tumor cells to the lung.^18^, ^19^, ^20^ Perhaps, this explains the concurrent metastasis to other distant secondary organs such as the lungs, brain, and liver in about two‐thirds of the patients.^19^ Concurrent detection of metastatic RCC at multiple sites has been enhanced following the advent of imaging technologies such as computed tomography (CT), magnetic resonance imaging (MRI), and fluoro‐2‐deoxy‐D‐glucose positron emission tomography (FDG).^19^

Cases of Guillain‐Barre syndrome (an acute immune‐mediated inflammatory peripheral neuropathy) developing in metastatic RCC patients treated with pazopanib and sunitinib malate have been reported.^21^, ^22^ In the present case, patient was diagnosed with Guillain‐Barre syndrome as a child prior to a diagnosis of a kidney mass made in 2014 as an adult. Notably, there was no history of treatment with sunitinib, or related drugs, prior to the diagnosis of Guillain‐Barre syndrome in the current case.

Although surgical excision of primary and metastatic lesions remains a standard procedure in the management of patients with RCC, there has been an increased insight into the molecular biology of RCC offering the potential for the development of new therapeutic strategies.^23^, ^24^, ^25^ Unfortunately, surgical treatment of RCC patients remains a palliative measure as over 90% of patients die within one year of diagnosis.^4^, ^23^, ^24^, ^25^

In conclusion, metastatic RCC to the oral cavity, because of its rarity, presents a diagnostic oddity. In the present case, the oral tumor was the initial sign and presentation of the disease prompting further investigation into the primary source and location. Clinicians should therefore increase their index of suspicion for solid oral mucosal lesion to include the possibility, though very rare, of metastatic RCC that may clinically masquerade as local and benign epulides of the oral mucosa. Prompt excision of such lesion and submission of the specimen for histopathologic examination should constitute a routine practice.

## CONFLICTS OF INTEREST

None declared.

## AUTHOR CONTRIBUTION

SP: involved in examination of patient and diagnosis, surgical treatment and follow‐up of patient, and preparation of draft of the manuscript. JB: involved in examination of patient and diagnosis, surgical treatment and follow‐up of patient, and preparation of draft of the manuscript. NN: involved in examination of patient and diagnosis, histopathologic diagnosis of surgical specimen, and preparation of draft of the manuscript. KO: involved in histopathologic diagnosis of surgical specimen, preparation of draft of the initial manuscript, review and correction of final manuscript, and submission of the manuscript.
